# Who Peeked? Children Infer the Likely Cause of Improbable Success

**DOI:** 10.1111/desc.13598

**Published:** 2024-12-20

**Authors:** Amy M. Chung, Terryn Kim, Ori Friedman, Stephanie Denison

**Affiliations:** ^1^ Department of Psychology University of Waterloo Waterloo Ontario Canada

**Keywords:** cheating detection, intentionality, probabilistic reasoning, theory of mind

## Abstract

Some outcomes are brought about by intentional agents with access to information and others are not. Children use a variety of cues to infer the causes of outcomes, such as statistical reasoning (e.g., the probability of the outcome) and theory of mind (e.g., a person's perceptual access, preferences, or knowledge). Here we show that children use these cues to infer cheating, a finding which informs our understanding of the flexibility of children's theory of mind. In four experiments (*N* = 444), 4‐ to 7‐year‐olds saw vignettes about blindfolded agents retrieving 10 gumballs from a distribution of yummy and yucky gumballs. Children were then asked if agents were really blindfolded or had peeked. We manipulated the probability of the outcome (i.e., the correspondence between the distribution sampled from and the outcome produced) and the ordering of the outcome was patterned (e.g., five yummy then five yucky) or haphazard. From age 5, children began to use both cues to infer cheating, and also showed signs of flexibly integrating these cues. Together, these findings show that young children can detect cheaters, and that their theory of mind reasoning is flexible and not based on simple and rigid rules (e.g., equating not‐seeing with failure). The findings also suggest that children use probabilistic reasoning to infer knowledge.

## 1 Introduction

Two children share a bag of gummy bears. They both like red ones best, but there are five different colors in the bag. They agree to each close their eyes and choose 10 bears from the bag, one at a time. The first child chooses: red–red–red–red–red–red–red–red–yellow–yellow. Maybe he was very lucky, but this looks suspicious! Given the *distribution* of gummy bears, it's unlikely that he randomly picked so many red ones. Moreover, this might seem especially unlikely because of the *order* in which he picked them—all the reds were in a row. At this point, the other child might accuse him of cheating—he probably peeked and saw which candies he was picking.

**Summary**

- We examine whether 4‐ to 7‐year‐olds use probability and ordering of outcomes when asked whether agents cheated.
- At age 5, children begin to use both probability and ordering of outcomes to infer cheating.
- Children show signs of integrating the cues and may prioritize probability over ordering.
- Our findings show children's theory of mind is flexible and not based on rigid rules about success and failure.

In this paper, we investigate if children recognize cheating by making these kinds of assessments about improbable distributions and orderings. By now, much work suggests that children's judgments are often informed by whether outcomes or selections deviate from chance expectations. For instance, when an agent repeatedly chooses one kind of toy from a container of toys, preschoolers do not rashly assume it is the agent's favorite (Kushnir, Xu, and Wellman 2010; also see Diesendruck et al. 2015; Flanagan et al. 2024; Ma and Xu 2011; Wellman et al. 2016). Instead, they consider the distribution of toys in the container and whether the agent chose that kind of toy more than would be expected by chance. For example, if there are many more toy ducks than frogs and the agent chooses ducks, she could have done so by grabbing them without much thought, but if she chooses frogs, then this signals an intention, perhaps driven by a preference. Children similarly consider departures from chance to inform the meaning of new words (Xu and Tenenbaum 2007), to anticipate others' emotions like surprise and happiness (e.g., Doan, Friedman, and Denison 2020; Wu, Merrick, and Gweon 2024), and to and infer social relationships and racial attitudes (Eason, Kaiser, and Sommerville 2019; Heck, Kushnir, and Kinzler 2021; Sehl, Friedman, and Denison 2023). For instance, they predict that agents will be surprised by improbable outcomes (Doan, Friedman, and Denison 2018) and that agents are likely to be friends with one another if the proportion of friends they have in common exceeds what would be expected by chance (e.g., Sehl, Friedman, and Denison 2023).

Existing work also suggests that children may see ordered outcomes as unlikely to result as a matter of mere chance. Children as young as 4 years reason that order is likely to be caused by human agents and unlikely to be caused by elements like the wind (Friedman 2001; see also Newman et al. 2010). In these experiments, children saw items such as red and green marbles that started out in a haphazard arrangement (colors intermixed) but appeared ordered after a delay (three rows of green, three rows of red). They reasoned that a person could have caused this change from disorder to order, but that the wind could not. Also, in infant looking time experiments, 9‐ and 10‐month‐olds expected that a human agent could create sequences of red and yellow balls that were patterned (e.g., repeating R–Y–Y five consecutive times) but that a mechanical device could only create non‐patterned sequences (Ma and Xu 2013).

Our investigation is informative about the flexibility of children's theory of mind. Some theorists posit that children reason about others’ minds using simple deterministic rules, such as ‘*seeing leads to knowing and success’* and ‘*not‐seeing leads to ignorance and failure*’ (e.g., Fabricius et al. 2021; Ruffman 1996; Saxe 2005). On such accounts, children should have difficulty understanding mixed success, as when someone gets both desirable and undesirable gummy bears from a bag. In particular, children should be insensitive to how often the person succeeds, and whether their successes and failures are ordered or haphazard. So, if children do use these considerations to infer knowledge, it means they are not limited to simple rules like ‘*not‐seeing leads to ignorance and failure*’. Instead, such findings would suggest a flexible inferential system where children seek to explain results that deviate from chance expectations by consulting their causal knowledge (e.g., theory of mind).

This investigation is also informative about how children detect cheating and deception. Many studies have investigated the circumstances under which children lie (e.g., Evans and Lee 2013; Talwar and Lee 2008) and cheat (e.g., Fu et al. 2016; Zhao et al. 2024), often using paradigms where children can only win a prize if they peek at the answer to a difficult question. However, only a few studies have examined children's ability to detect deception in others (e.g., Ghossainy, Al‐Shawaf, and Woolley 2021; Lee et al. 2002; Levush and Butler 2024), but they did not look at children's use of statistical reasoning (but see Oey, Schachner, and Vul 2022 for a relevant study on adults).

Our experiments contribute by examining whether children use statistical reasoning to decide whether agents cheated when explicitly asked about this (i.e., we do not examine children's spontaneous inferences of cheating).

To explore how children infer cheating from improbable distributions and orderings, we showed children stories where blindfolded agents picked several gumballs from a bowl containing both yummy red gumballs and yucky purple ones. We asked children whether the agents had peeked (following Aboody, Huey, and Jara‐Ettinger 2022) to test whether they thought agents knew which gumballs they were choosing and had thus cheated. Some agents retrieved ratios of red to purple gumballs that corresponded with the ratio of these gumballs in the bowl whereas other agents retrieved advantageous ratios with more red gumballs than would be expected by chance. Also, for some agents the order in which the gumballs were retrieved looked random (e.g., two reds, then five purples, then three more reds), while for others the order was more structured (e.g., five reds followed by five purples).^1^

To start, in Experiment 1, we looked at both cues together to see if children infer cheating when it is especially obvious. For each agent, the ratios and patterning structure were either both suspicious (i.e., improbably good and ordered) or indicative of randomness. In subsequent experiments, we manipulated them independently.

## 2 General Methods and Analytic Approach

The materials, data, and code from all experiments are available online at https://osf.io/a7nge/. Most children were tested in person at their child‐care centers and schools (54% in Experiment 1, 83% in Experiment 2, and 94% in Experiment 3) and the rest were tested remotely using Zoom. Demographic information was not collected from each child (as per allowances of our IRB application). However, 64% of residents in the region are White; South Asians are the largest visible minority. In all experiments, we aimed to test 20 children at each age in years per between‐subject group, but we raised this to 30 in Experiment 2 because it had an additional factor.

We analyzed results using generalized estimating equations (GEE) models run in R using ggpack (Højsgaard, Halekoh, and Yan 2006), entering manipulated factors and age (in months) as predictors. These models analyze repeated measures data without averaging or collapsing responses across trials. We used GEEs because they yield similar results to mixed models without the need to add random intercepts and slopes (see Frank et al. 2025). We used *emmeans* (Lenth et al. 2023) to derive Type III omnibus tests and for post‐hoc comparisons. We used *ggeffects* (Lüdecke 2018) to plot the models and to examine 95% confidence intervals (CI). Examining confidence intervals provides a conservative estimate of when responses across conditions first differ, and when they first depart from chance. Although our analyses focus on children's ages in months, the Supporting Information provides tables summarizing the results broken down by age in years.

## 3 Experiment 1

### 3.1 Method

#### 3.1.1 Participants

We tested eighty 4‐ to 7‐year‐olds (*M*_age_ = 6;0, range = 4;0–7;10, 44 girls and 36 boys) with 20 children at each age in years. Two additional 4‐year‐olds were tested and excluded because they responded with colors when asked whether the agent peeked or not.

#### 3.1.2 Materials and Procedure

**FIGURE 1:**
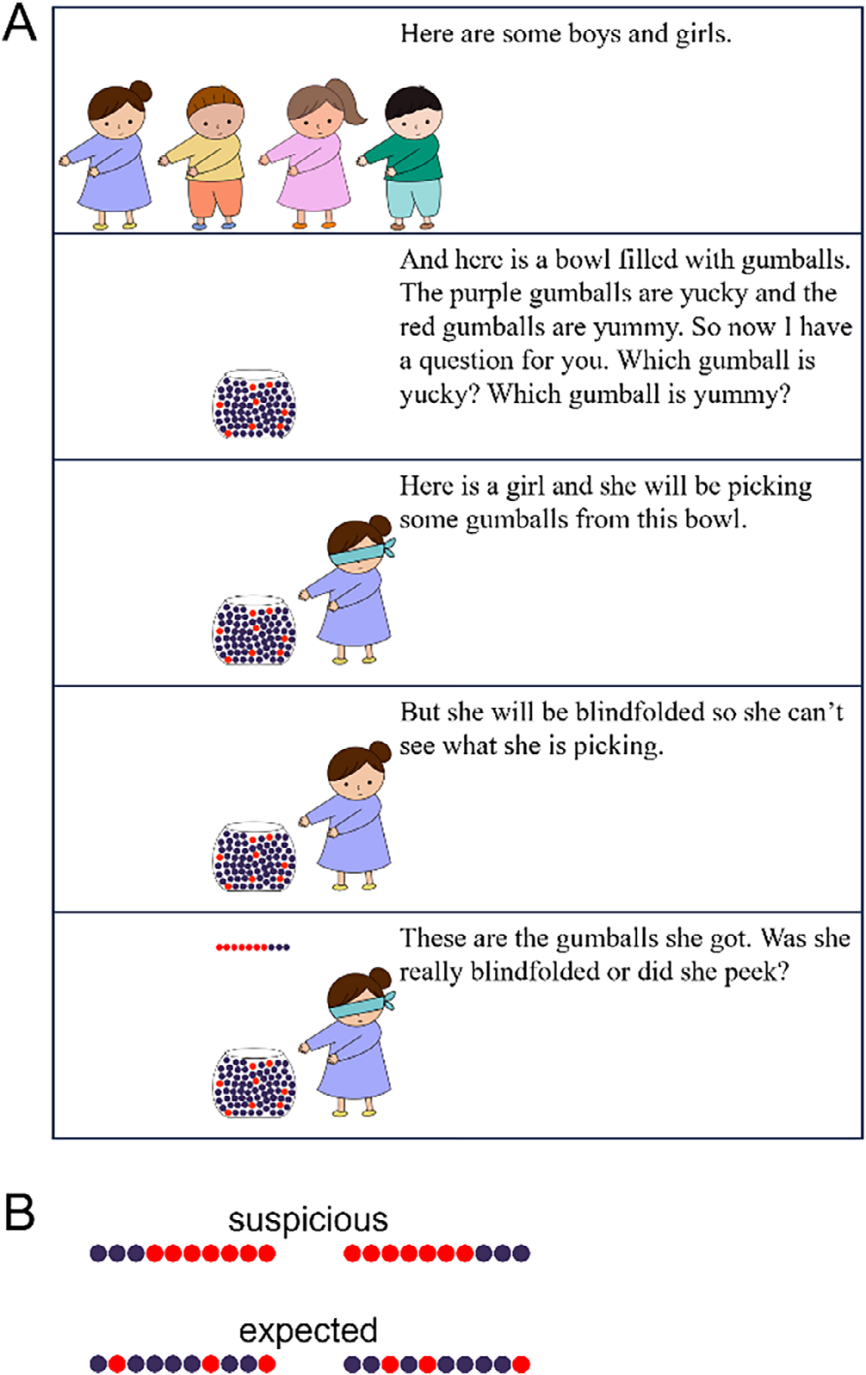
Experiment 1: Sample slides and script. Each child completed four trials in a within‐subjects design where agents retrieved gumballs from a bowl offering poor odds. The outcomes were either suspiciously good (two trials) or as expected (two trials). Panel A shows the introduction and one sample trial (suspicious outcome). Panel B shows the gumballs in the outcomes across the four trials.

Children first saw four characters and a bowl containing many yucky purple gumballs and a few yummy red ones (66 purple and 8 red). To confirm children understood, they were asked which gumballs were yucky and which were yummy; see Figure 1 for the script and accompanying slides.

Next, children completed four test trials. In each trial, a different character picked 10 gumballs from the bowl while appearing to be blindfolded. The characters were each faced with the same distribution of gumballs, but they produced different outcomes. In two trials, the character produced an expected outcome: seven yucky gumballs and three yummy ones, and these appeared randomly ordered. In the other two trials, the outcome was suspiciously good: seven yummy and three yucky ones and the ordering was systematic (e.g., seven yummy in a row, followed by three yucky). At the end of each trial, children were asked whether the character had peeked (e.g., “Was she really blindfolded or did she peek?”). Children saw trials in either of two orders: suspicious, expected, expected, suspicious or expected, suspicious, suspicious, expected.^2^

### 3.2 Results and Discussion

**FIGURE 2:**
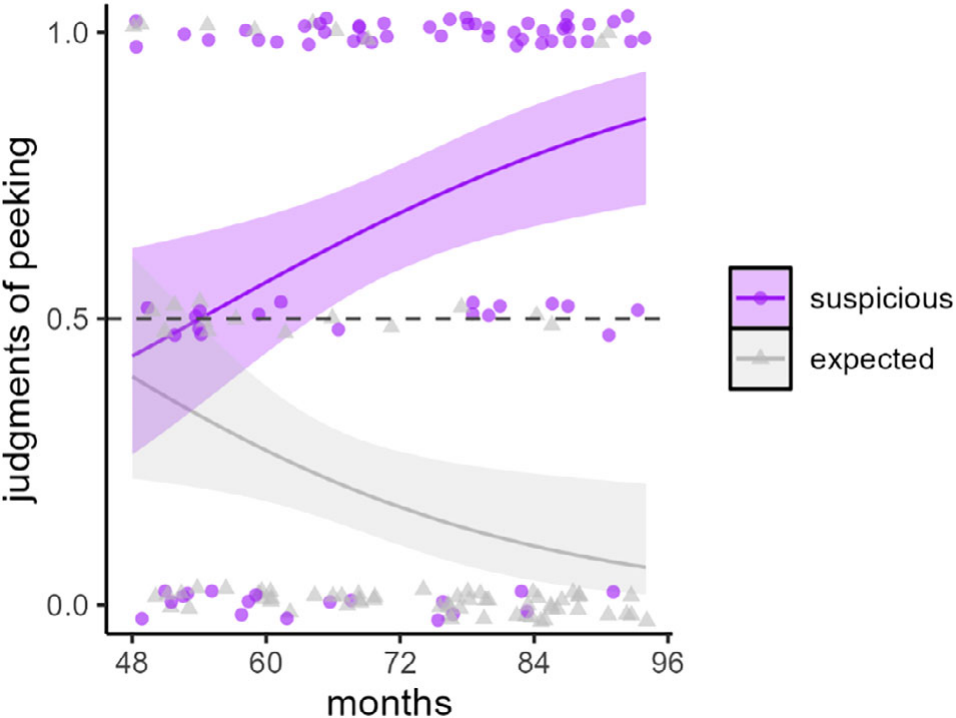
Peeking judgments in Experiment 1. Children saw scenarios where agents’ choices were either suspiciously good or as expected and judged whether each agent peeked (1) or remained blindfolded (0). In all plots, the line shows the output of the GEE model and bands show 95% CIs; jittered points show individual participant's responses averaged across trials.

We entered responses of peeking (scored 1) and blindfolded (scored 0) into a GEE model with outcome (expected, suspicious) and age (in months) as predictors; see Figure 2. The model revealed a main effect of outcome, *χ* ^2^(1) = 34.91, *p* < 0.001, no main effect of age, *χ* ^2^(1) = 0.05, *p* = 0.832, and a significant interaction between these factors, *χ* ^2^(1) = 10.51, *p *= 0.001.

Overall, children were more likely to say that characters peeked if their outcomes were suspicious rather than expected. The interaction with age resulted because in comparison with younger children, older children were more likely to say agents with suspicious outcomes had peeked, *χ* ^2^(1) = 7.50, *p* = 0.006, and less likely to say this about agents with expected outcomes, *χ* ^2^(1) = 5.04, *p* = 0.025. To determine the age where responses first differed across the expected and suspicious outcome conditions, we examined when their confidence intervals no longer overlapped. This was at age 4;11 (59 months): suspicious outcome, CI_95%_[0.43, 0.67]; expected outcome [0.19, 0.40]. Examining confidence intervals also suggested that children mostly attributed cheating for suspicious outcomes from age 5;5 (65 months), CI_95%_[0.51, 0.71], and mostly denied cheating for expected outcomes from age 4;6 (54 months), CI_95%_[0.20, 0.49].

These findings suggest that from age 5, children infer cheating when characters produce suspicious outcomes, as compared to expected ones. In this experiment, though, two cues worked in tandem to make outcomes suspicious—they were improbably good and ordered. In Experiment 2, we attempt to tease these apart by presenting outcomes in varying degrees of probability (20%, 50%, and 80% yummy) and crossing this with ordering (structured or random). This resulted in six different combinations.

## 4 Experiment 2

### 4.1 Method

#### 4.1.1 Participants

We tested one hundred twenty‐three 4‐ to 7‐year‐olds (*M*_age_ = 5;11, range = 4;0–7;11, 72 girls and 51 boys). We intended to test 30 children at each age in years, but also included three extra participants—two extra 5‐year‐olds and one extra 7‐year‐old. Also, data from one further child was excluded because they did not respond on multiple trials.

#### 4.1.2 Materials and Procedure

**FIGURE 3:**
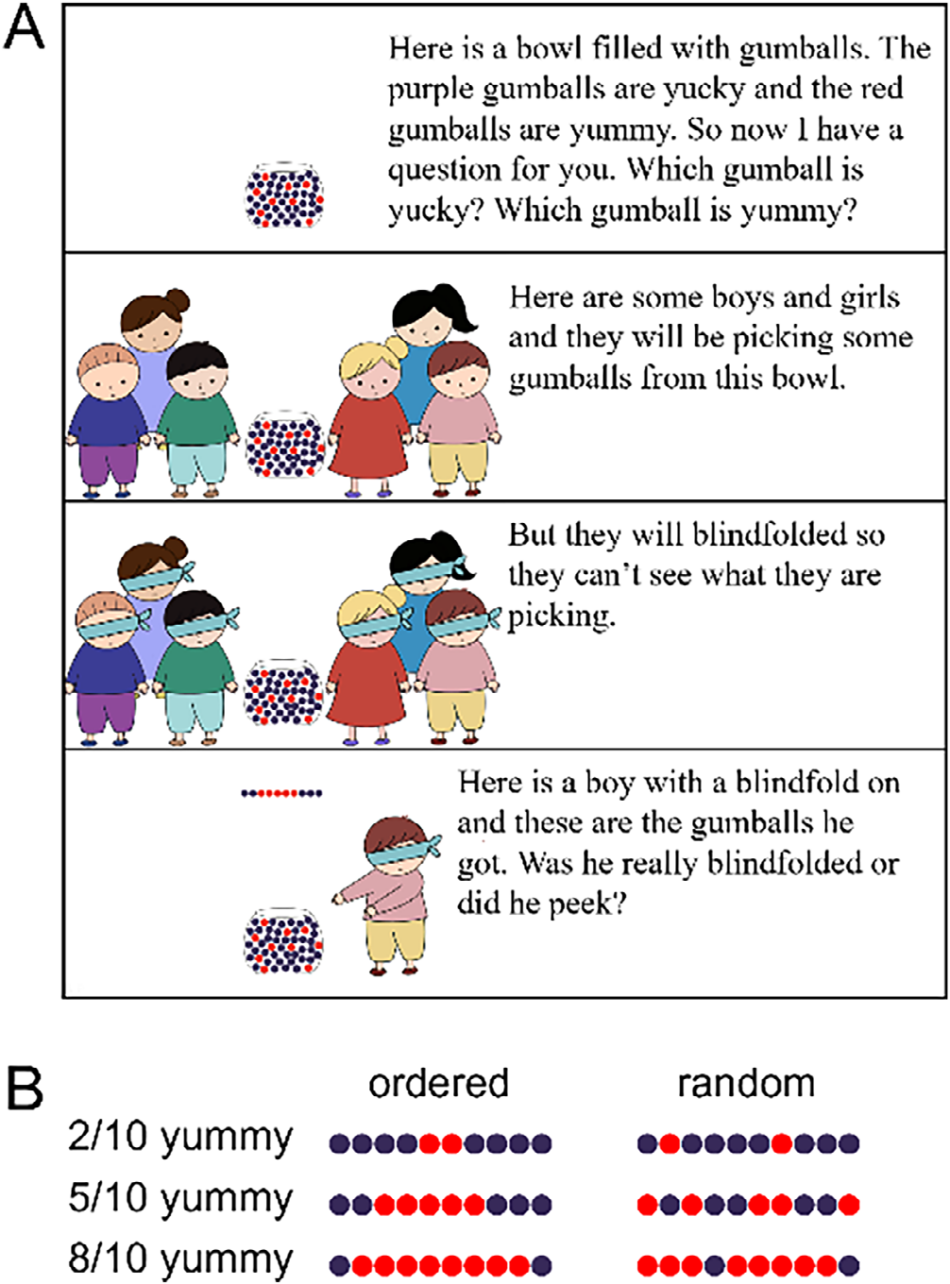
Experiment 2: Sample slides and script. Each child completed six trials in a within‐subjects design where agents retrieved 2, 5, or 10 yummy gumballs from a bowl offering poor odds, and the ordering of retrieval was either ordered or random. Panel A shows the introduction and a sample trial (5/10 ordered). Panel B shows the gumballs in the outcomes across the six trials.

Children were first told about a bowl with mostly yucky purple gumballs and some yummy red ones (40 purple and 10 red); see Figure 3 for the testing script and sample slides. After responding to comprehension questions confirming they knew which gumballs were which, they were told that six characters would choose 10 gumballs from the bowl while blindfolded. Each of these six characters served as one cell in a 3 × 2 within‐subjects design manipulating whether the ratio of yummy gumballs retrieved was low, high, or medium (i.e., 2/10, 5/10, or 8/10), and whether the order of retrieval was structured (e.g., all yummy, then all yucky) or random. As before, children were asked whether each character peeked or was really blindfolded.

### 4.2 Results and Discussion

**FIGURE 4:**
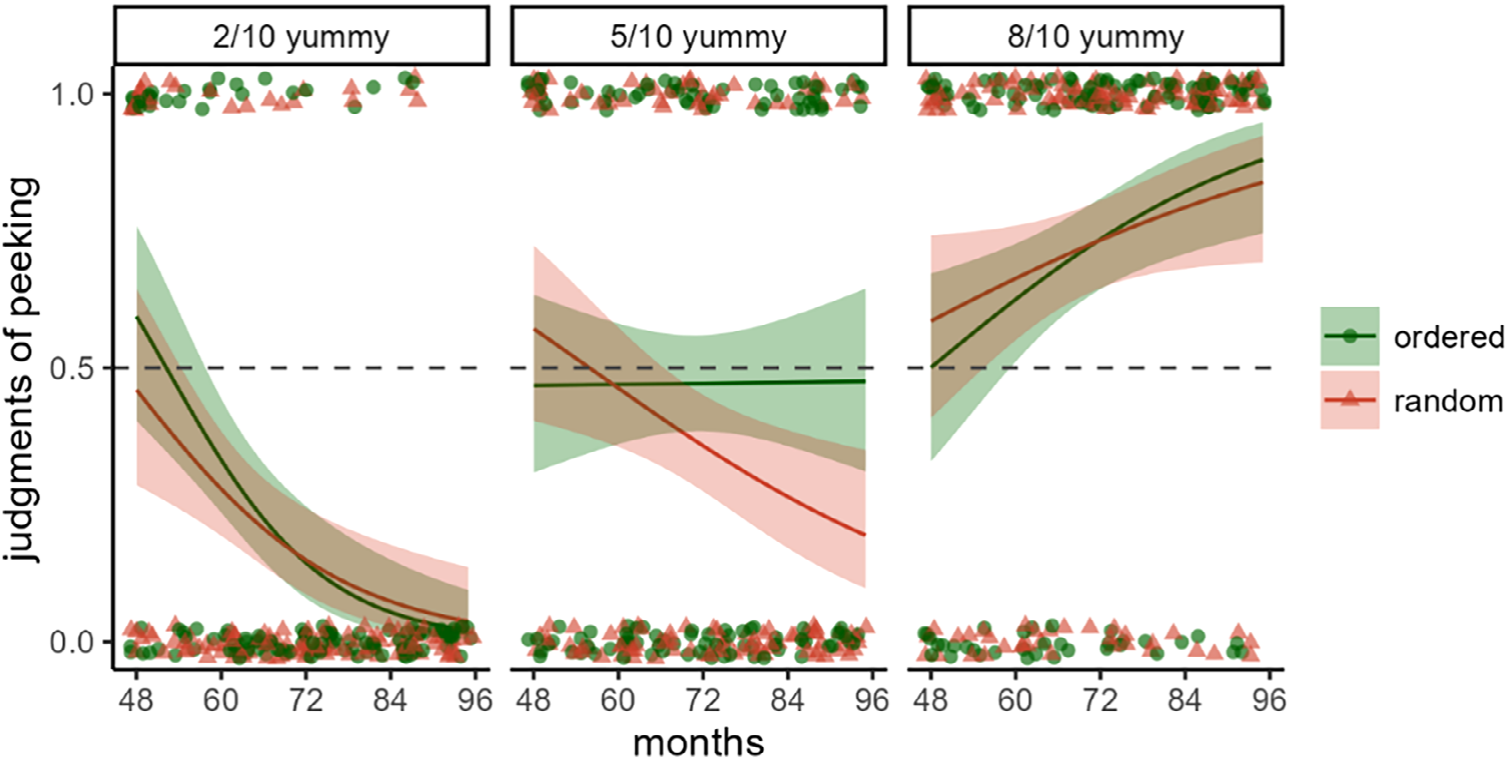
Peeking judgments in Experiment 2. Children saw scenarios where agents retrieved a low, high, or medium (i.e., 2/10, 5/10, or 8/10) proportion of yummy gumballs from a bowl with mostly yucky ones, and where the order of retrieval was ordered or random. Children judged whether each agent peeked (1) or remained blindfolded (0).

We entered responses of peeking (scored 1) and blindfolded (scored 0) into a GEE model with ratio (2/10, 5/10, and 8/10 red gumballs), ordering (ordered, random), and age as predictors; see Figure 4. The model revealed a significant main effect of ratio, *χ* ^2^(2) = 78.70, *p* < 0.001, but no main effect of ordering, *χ* ^2^(1) = 1.22, *p* = 0.270, or age, *χ* ^2^(1) = 2.93, *p* = 0.087. There was a significant 2‐way interaction between ratio and age, *χ* ^2^(2) = 32.97, *p* < 0.001, and the 3‐way interaction was also significant, *χ* ^2^(2) = 7.33, *p* = 0.026. The other interactions were non‐significant: ratio × ordering, *χ* ^2^(1) = 3.18, *p* = 0.204, and ordering x age, *χ* ^2^(1) = 1.29, *p* = 0.256.

To follow up on the significant interactions, we ran separate ordering x age analyses for each ratio; see Table 1. Ordering did not matter when the agent received a low (2/10) or high (8/10) ratio of yummy gumballs. At these ratios, the only significant result was a main effect of age. When the ratio of yummy gumballs was low, older children increasingly denied the agent had peeked, *p* < 0.001; when the ratio was high, they increasingly said the agent peeked, *p* = 0.010. By contrast, when the agent received a 5/10 ratio of yummy gumballs, there was a main effect of ordering, *p* = 0.040, and it also interacted with age, *p* = 0.009. As can be seen in Figure 4, this interaction resulted because there was no effect of age when the outcome was ordered, *χ* ^2^(1) = 0.00, *p* = 0.960, but there was a decline in judgments of peeking when the outcome was random, *χ* ^2^(1) = 6.93, *p* = 0.009. We also examined the age at which children's responses differed across the different ratios and across the two orders of the medium ratio. At 4 years 9 months, children were more likely to judge peeking for the character with a high ratio of yummy gumballs, CI_95%_[0.50, 0.73] than a low ratio CI_95%_[0.26, 0.47], and at 5 years 2 months, their responses differed for all three levels: high, CI_95%_[0.56, 0.75]; medium, CI_95%_[0.37, 0.55]; and low, CI_95%_[0.20, 0.36]. Turning to ordered and random within the medium ratio, we did not find an age where their confidence intervals did not overlap.

**TABLE 1:** Effects of ordering and age at each ratio (low, medium, and high) in Experiment 2.

	Low (2/10)		Medium (5/10)		High (8/10)	
Effect	*χ* ^2^(1)	*p*	*χ* ^2^(1)	*p*	*χ* ^2^(1)	*p*
Ordering	0.00	0.953	4.20	0.040	0.00	0.968
Age	15.11	0.001	2.53	0.112	6.65	0.010
Ordering × age	1.78	0.182	6.80	0.009	1.51	0.220

Finally, we also looked at confidence intervals to determine the ages at which children mostly attributed cheating and mostly denied it. With a low ratio of yummy gumballs, children mostly denied cheating starting at age 4;8 (56 months), CI_95%_[0.27, 0.49]; with a high ratio, they mostly affirmed cheating at 4;10 (58 months), CI_95%_[0.51, 0.73]. With a medium ratio, the results must be split by ordering given the 3‐way interaction. With ordered gumballs, responses did not vary with age and do not depart from chance, CI_95%_[0.39, 0.56]; with the random ordering, children mostly denied cheating from 5;7 (67 months), CI_95%_[0.31, 0.49].

These results suggest that before they are 5, children are more likely to conclude agents peeked when they produced improbably good outcomes rather than worse ones. The findings also suggest that children use ordering to infer peeking under specific circumstances—when the ratio of yucky to yummy gumballs is only slightly better than chance (5/10 from a 20% distribution), and not otherwise. This could reflect a process where children first consider ratio information, and only supplement it with information about ordering when ratio information is not decisive. However, the findings are unclear about the ages at which children are sensitive to ordering: Although ordering and age interacted for the 5/10 ratio, examining the confidence intervals did not provide a clear indication of when responses across the orderings differ. To determine this, in the next experiment we attempted to use a more sensitive approach to looking at ordering of outcomes in 5‐ to 7‐year‐olds. The experiment isolates ordered versus random ordering using a forced‐choice paradigm.

## 5 Experiment 3

### 5.1 Method

#### 5.1.1 Participants

We tested 121 children aged 5–7 years old (*M*_age_ = 6;5, range = 5;0‐7;11, 64 girls and 57 boys). We intended to test 40 children per age, but also tested one extra 5‐year‐old.

#### 5.1.2 Materials and Procedure

**FIGURE 5:**
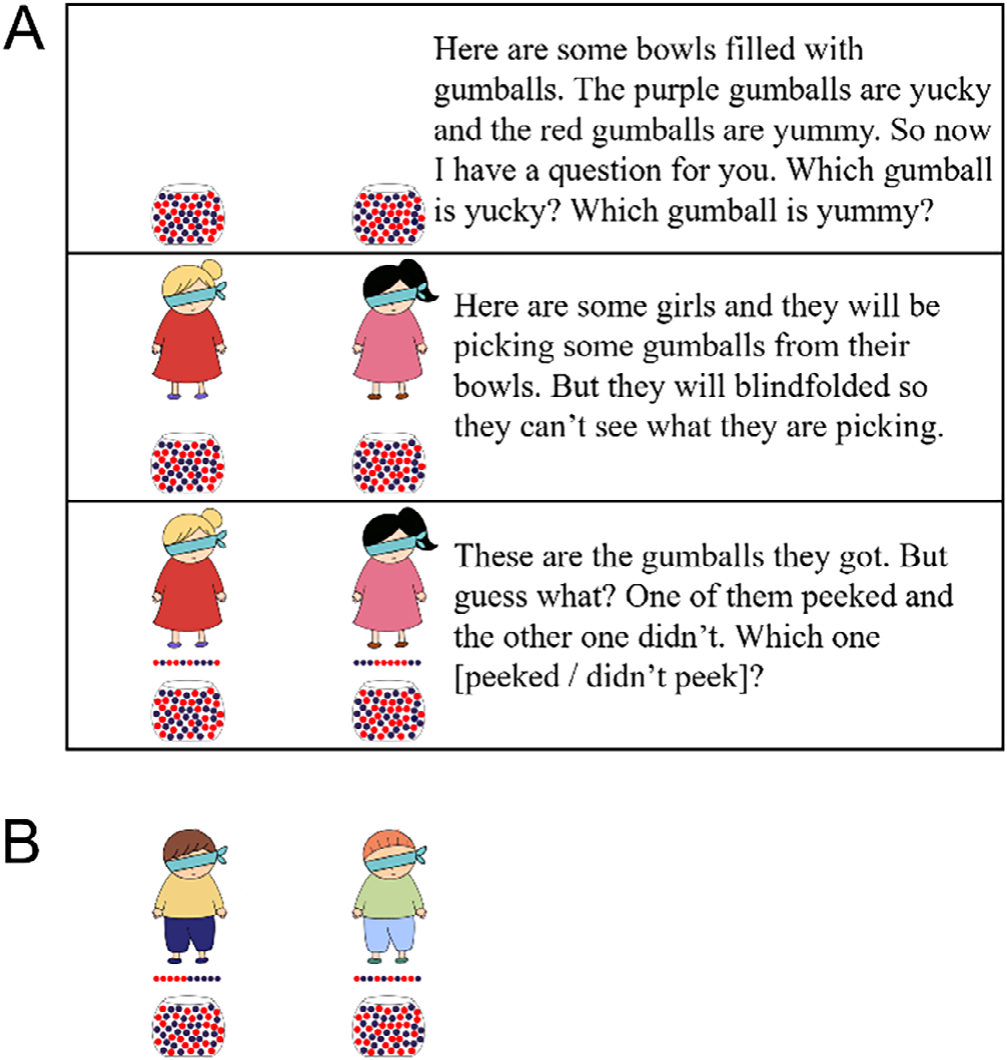
Experiment 3: Sample slides and script. Each child completed two trials in a between‐subjects design manipulating whether participants were asked about which agent peeked or about which did not peek. In each trial, the order of retrieval was ordered for one agent and random for the other. Panel A shows the introduction and the first trial (vignette about two girls); text in square brackets varied across between‐subjects conditions. Panel B shows the final slide in the second trial (vignette about two boys).

Children were first shown two bowls with gumballs. Half the gumballs in each bowl were yucky purple ones and the other half were yummy red ones; see Figure 5 for the script and sample slides. Children were asked comprehension questions confirming they understood which gumballs were which, and then saw two vignettes.

Each child then saw two vignettes. In both vignettes, two characters wearing blindfolds each stood near one bowl and took 10 gumballs from it. Both characters received five yucky gumballs and five yummy ones. However, one character's gumballs were arranged randomly whereas the other character's gumballs were ordered. At the end of each vignette, children were told that one character peeked and the other did not. They were then asked a test question, which varied across between‐subject conditions. In one condition, children were asked which character peeked (“Which one peeked?”; in the other, they were asked which character didn't peek (“Which one didn't peek”).^3^

The first vignette was about two girls and the second one was about two boys. Also, children either saw vignettes where the ordered gumballs were on the left in the vignette with girls and on the left in the one about boys, or vignettes where this was reversed.

### 5.2 Results and Discussion

**FIGURE 6:**
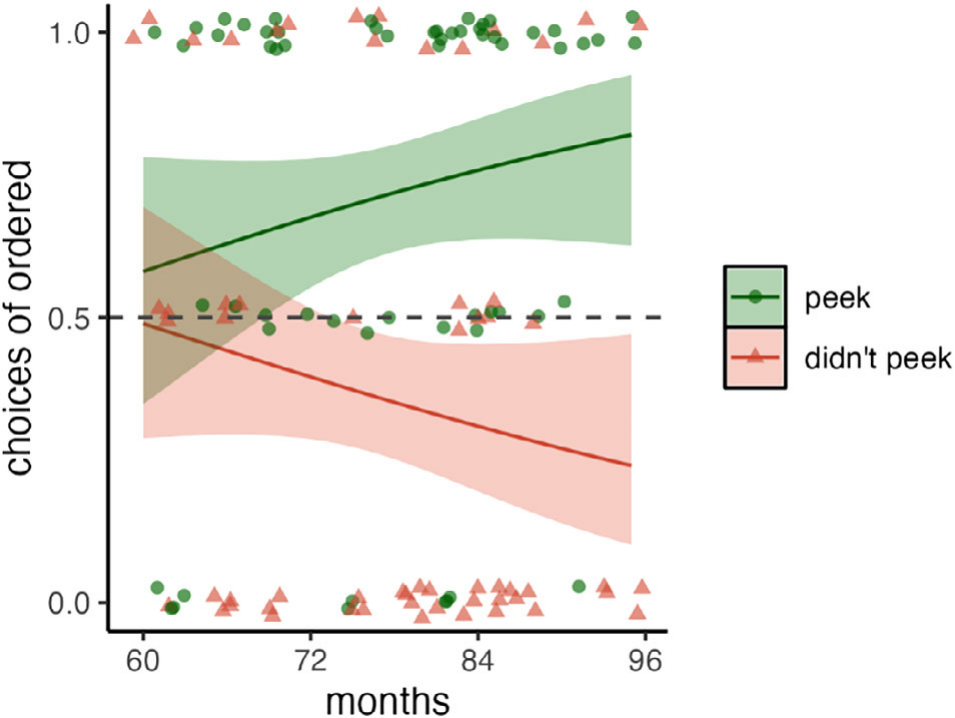
Choices of the character with ordered gumballs in Experiment 3. Children saw scenarios where two agents retrieved yummy and yucky gumballs, but where the order of retrieval was ordered for one agent and random for the other. Children judged which agent had peeked: the agent with the ordered sequence (1) or the random sequence (0).

We entered choices of the agent with the ordered outcome (scored 1) and random outcome (scored 0) into a GEE model with question (peeked, did not peek) and age as predictors; see Figure 6. The model revealed a main effect of question, *χ* ^2^(1) = 20.14, *p* < 0.001, but no main effect of age, *χ* ^2^(1) = 0.01, *p* = 0.944, and no interaction, *χ* ^2^(1) = 3.57, *p* = 0.059. The main effect resulted because children mostly chose the character with ordered gumballs when asked who peeked, CI_95%_[0.61, 0.80] and mostly chose the character with random ones when asked about who did not peek, CI_95%_[0.26, 0.47].

These findings confirm that 5‐ to 7‐year‐olds use outcome order to infer cheating. The findings of Experiment 2 also suggested this, but the effect of ordering was somewhat unreliable (i.e., it only turned up for one level of probability and even there the 95% confidence intervals overlapped across ordered and random outcomes at all ages). Here, we used a forced‐choice question about which agent peeked, and the results were clearer.

In our final experiment, we return to children's use of ratios to infer cheating. In the earlier experiments, we manipulated the ratio of yucky to yummy gumballs that the agent retrieved, while keeping the distribution in the bowl constant for all agents. In those experiments, children suspected peeking when agents retrieved a high proportion of yummy gumballs but not when they retrieved a low proportion. Although this suggests that children used statistical reasoning to infer whether outcomes were suspiciously good, another possibility is that children used a low‐level heuristic: Whenever agents get many yummy gumballs, suspect cheating regardless of whether this is probable based on the ratio in the bowl. In our final experiment, we addressed this possibility by varying the ratio in the bowl from which the gumballs were drawn.

## 6 Experiment 4

### 6.1 Method

#### 6.1.1 Participants

We tested one hundred twenty 5‐ to 7‐year‐olds (*M*_age_ = 6;3, range = 5;0–7;11, 61 girls, 58 boys, and one child whose gender was not disclosed). We again intended to test 40 children per age. Data from two additional children were excluded—one child did not respond to the test questions and the other child repeatedly failed a comprehension question.

#### 6.1.2 Materials and Procedure

**FIGURE 7:**
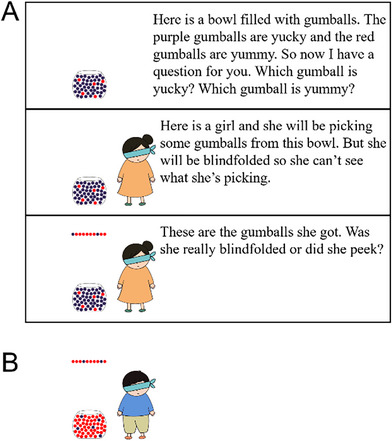
Experiment 4: Sample slides and script. Each child completed two trials in a between‐subjects design manipulating whether gumballs were retrieved from a bowl offering good or bad odds. Panel A shows the introduction and first trial (vignette about a girl) from the between‐subjects condition where agents retrieved gumballs from a bowl offering bad odds. Panel B shows the final slide from the second trial (vignette about a boy) from the other between‐subjects condition the bowl offered good odds.

Children were first told about a bowl with yucky purple gumballs and yummy red ones; see Figure 7 for the testing script and sample slides. The bowl either contained mostly yucky gumballs or it mostly contained yummy ones (manipulated between‐subjects; 45 of the majority color and 5 of the minority color). After responding to comprehension questions confirming they knew which gumballs were which, children saw two stories. In each story, a blindfolded agent chose 10 gumballs from the bowl. Of these, eight were yummy, and children were asked if the agent had really been blindfolded or whether they had peeked. In the first story, the agent was a girl, and in the second it was a boy.

### 6.2 Results and Discussion

**FIGURE 8:**
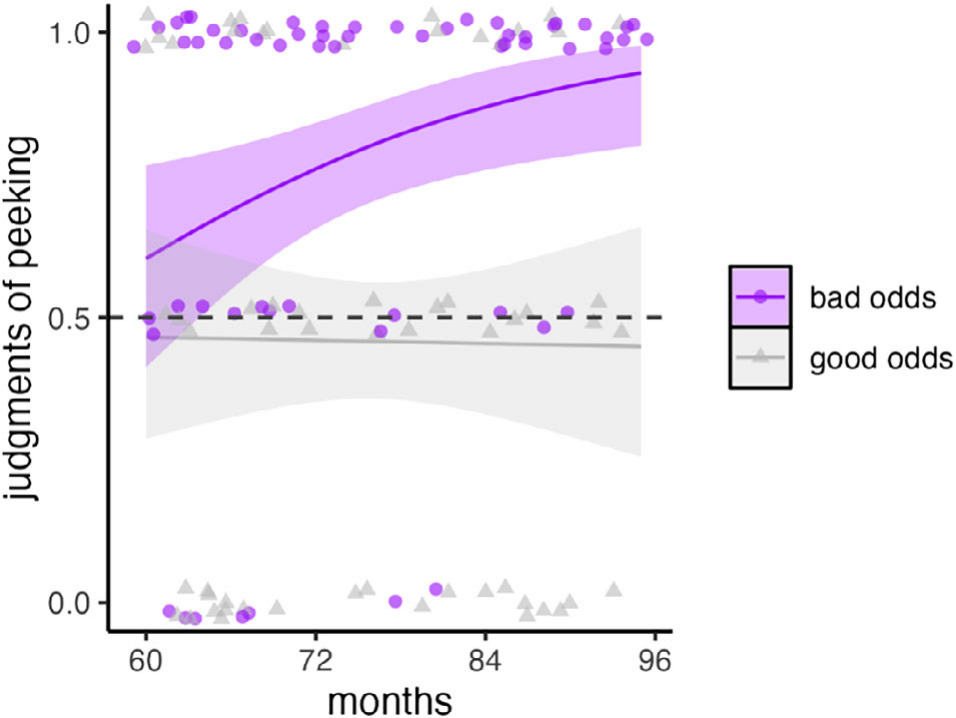
Peeking judgments in Experiment 4. Children saw scenarios where agents retrieved a high proportion of yummy gumballs from a bowl which either offered good or bad odds of getting yummy gumballs. Children judged whether each agent had retrieved gumballs by peeking (1) or remaining blindfolded (0).

We entered judgments of peeking (scored 1) and blindfolded (scored 0) into a GEE model with condition (bad odds and good odds) and age as predictors; see Figure 8. The model revealed a significant main effect of condition, *χ* ^2^(1) = 19.67, *p* < 0.001, no main effect of age, *χ* ^2^(1) = 3.59, *p* < 0.058, and a significant interaction between condition and age, *χ* ^2^(1) = 4.05, *p* = 0.044.

Overall, children were more likely to say that agents peeked if they obtained a good outcome from a bowl offering bad odds than from one offering good odds. The interaction with age resulted because older children were more likely than younger ones to infer peeking for the character with bad odds, *χ* ^2^(1) = 6.63, *p* < 0.010, whereas responses did not change with age for the character with good odds, *χ* ^2^(1) = 0.01, *p* = 0.928. Responses across the two conditions first differed at age 5 years; 9 months: bad odds, CI_95%_[0.61, 0.82]; good odds, CI_95%_[0.34, 0.59]. Also, children mostly attributed peeking for bad odds starting at age 5;4 (64 months), CI_95%_[0.51, 0.79].

Thus, children's peeking judgments vary for identical outcomes depending on the distributions from which they are drawn, showing that they consider the probability of the outcome and not just whether it has mostly good items. This shows that children were not reasoning using the low‐level heuristic of inferring cheating whenever agents get many yummy gumballs. It's true that the youngest children's responses did not differ across the bad and good odds conditions. Even so, these children inferred peeking at chance, which also does not fit with the heuristic—if young children had heeded it, they should have attributed cheating at high rates in both conditions.

## 7 General Discussion

In four experiments we examined whether children use probability and ordering to infer cheating by asking whether an agent peeked while selecting items. We found that by age 5, children are more suspicious of outcomes that are improbably good, and are more suspicious of ordered outcomes than disordered ones. These findings suggest that children use statistical considerations to recognize cheating, consistent with a large literature showing that children use statistical information to make many social inferences (Diesendruck et al. 2015; Doan, Friedman, and Denison 2018, Doan, Friedman, and Denison 2020; Eason, Kaiser, and Sommerville 2019; Flanagan et al. 2024; Heck, Kushnir, and Kinzler 2021; Kushnir, Xu, and Wellman 2010; Ma and Xu 2011; Sehl, Friedman, and Denison 2023; Wellman et al. 2016; Wu, Merrick, and Gweon 2024). Thus, they contribute to our understanding of children's probabilistic reasoning, theory of mind, and cheating detection.

Before discussing the implications of the findings, we should acknowledge two important caveats. One is that although we often found success starting by age 5, the developmental patterns we observed were often protracted. So, although children first showed signs of using probability and ordering to detect cheating at age five, development continues and future work will be needed to understand why. The other caveat is that in our experiments, the possibility of cheating was pointed out to children in the test questions. This means the questions may have prompted children to think about cheating in situations where this would not have spontaneously occurred to them. Because of this, the findings are uninformative about how children recognize cheating when unprompted, as will usually be true in their regular lives. Instead, the main import of this work is to show that children are introduced to the possibility of cheating, they do not attribute it indiscriminately, at chance, or by using low‐level heuristics. Instead, they infer that agents have cheated by considering both the probability of success and failure and the ordering in which these occur.

### 7.1 Theory of Mind

Our findings suggest that children's theory of mind reasoning is flexible, and not based on simple and rigid rules like ‘seeing leads to knowing and success’ and ‘not‐seeing leads to ignorance and failure’ (e.g., Fabricius et al. 2021; Ruffman 1996; Saxe 2005). If children were limited to using these rules, they would have to conclude that agents peeked for all the yummy gumballs they retrieved, and did not peek for all yucky gumballs. But, if children saw things that way, they should have affirmed peeking for every agent. After all, each agent retrieved some yummy gumballs. Children's responses in our final experiment likewise show they did not use a simple heuristic of affirming cheating whenever agents had mostly good outcomes and denying it whenever agents had mostly bad ones. Importantly, we did not see evidence for these heuristics in the youngest children tested, even at ages where children responded at chance and did not use probabilistic information and ordering to infer cheating. This suggests that the developmental changes we observed did not involve a shift away from low‐level heuristics. Instead, it looks like the youngest children did not know which information to bring to bear when assessing cheating, and with age they increasingly used probabilistic information and ordering.

Taken together, the findings instead suggest that children expect uninformed (non‐peeking) agents to perform about as would be expected by chance—whether that expectation leads to a mostly good or mostly bad outcome. This requires thinking about the distribution of gumballs available and the odds of retrieving them, which is indicative of flexible integration of theory of mind and probability and is broadly consistent with infant work showing flexible integration of probability, naïve physics, and psychology (Attisano and Denison 2020; Denison et al. 2014; Téglás et al. 2007; Xu and Denison 2009).

Our findings may also speak to flexibility of theory of mind in another way. Children in the second experiment were sensitive to the ordering of the gumballs when the sample retrieved by the agent was middling (i.e., 5/10). Here, children were more likely to infer cheating when the gumballs were ordered (e.g., five yummy ones, followed by five yucky ones) than when they appeared random. By contrast, children were insensitive to ordering when the agent retrieved a sample that was extremely improbable (i.e., much better than expected by chance) and when it was entirely probable (exactly as poor as would be expected by chance). This may suggest that in detecting intentionality behind the sampling process, children flexibly integrated both kinds of information—they used probability alone when it provided strong evidence, but turned to ordering when the probabilistic information was not decisive. Admittedly, this finding could be explained in another way. The ordering of the retrieved gumballs might have been more obvious when there were equal numbers of yummy and yucky gumballs than when one kind was much more common than the other.

### 7.2 Knowledge

Beyond flexibility, the findings may also reveal a novel way that children infer knowledge from evidence. Specifically, our findings suggest that children inferred whether agents *knew* which gumballs they were selecting (or else were unaware of this) by considering proportional information and ordering. Previous work has shown that children use many cues to infer knowledge. One kind of cue children use is others’ access to information. For instance, 3‐ and 4‐year‐olds recognize that people know about hidden objects they recently saw or were told about (e.g., Pratt and Bryant 1990; Wimmer et al. 1988; Woolley and Wellman 1993). Children also infer knowledge based on agents’ past successes and failures. For instance, children attribute more knowledge to informants if they name objects correctly rather than incorrectly (Brosseau‐Liard and Birch 2010; Kushnir and Koenig 2017) or if they respond accurately without external help (Aboody, Huey, and Jara‐Ettinger 2022; also see Einav and Robinson 2011). Besides these cues, children also infer knowledge by considering cultural grouping (Soley 2019), social relationships (Liberman et al. 2020), and the nature of the information itself—for instance, whether it is generic or specific (Cimpian and Scott 2012). Our findings suggest that proportional information and ordering can be added to this list of cues.

One caveat to acknowledge, though, is that our task did not necessarily require children to think about knowledge per se—they could have considered the agent's perception alone. To focus on knowledge more directly, future work could explore judgments about situations where peeking guides later behavior (that occurs after the peeking ceases). For example, imagine an agent can pick 5 of 10 boxes, where only half the boxes contain rewards. If the agent chooses four boxes with rewards, we might wonder if the agent peeked ahead of time and therefore *knew* what was in each box (see Aboody, Huey, and Jara‐Ettinger 2022 for a related non‐probabilistic task).

### 7.3 Detecting Cheaters

Our findings suggest children are able to detect cheating in others, to the extent that peeking in a game counts as a form of cheating or deception. One prior study which looked at children's ability to detect deception may also have had a probabilistic element (Lee et al. 2002). In that work, children judged that a girl was lying to her mother about how a glass broke when the explanation offered by the girl was wildly improbable—she said a ghost from a picture book broke it. However, it could be that rather than thinking of this in terms of probability, children instead relied on the binary distinction between possible and impossible.

Future work could further investigate children's use of probability to detect cheating in tasks that have nothing to do with peeking. For example, if an agent rolls six with a die many times in a row, we may conclude they are cheating even though this has nothing to do with knowledge or perceptual access—maybe the agent is using a rigged die. Similarly, if an agent *claims* to have rolled six many times in a row, we may conclude they are lying. Recent work suggests that adults detect lying using probability in this way (Oey, Schachner, and Vul 2022). In these experiments, participants played a game where they reported the result of blind draws of balls from distributions with different probabilities of returning a winning ball. They found that adults suspect lying in others more often when the reported outcome is statistically unlikely rather than likely (e.g., they suspect lying more when a person reports receiving mostly winning balls from a distribution with only 20% winners as opposed to 80% winners). Similarly, when constructing lies, people fabricate outcomes that are probable, presumably to make their lies believable (Oey, Schachner, and Vul 2022). But surely children and adults do not only consider how believable or detectable their deception is when considering whether to lie or cheat (or whether to accuse someone else of such behavior).

A further extension of this work would be to explore the moral side of children's peeking or other judgments of deception. For example, to confirm whether children saw the agents who they deemed to have peeked as having done something wrong, and as deserving of punishment and how this impacts their willingness to accuse them. Finally, future work could also look at the development of children's spontaneous inferences of cheating—the circumstances in which children judge that others have cheated when the topic of cheating is not explicitly introduced to them.

## 8 Conclusion

Our findings reveal a novel way that statistical information impacts children's social judgments. From around age 5, children begin to use probabilistic information and ordering to recognize whether agents cheated by peeking. They use both cues in isolation and may prioritize probability when the cues are manipulated jointly. These abilities would not be possible if children equated ignorance with failure. So beyond showing how children recognize cheating, our findings demonstrate flexibility in their theory of mind.

## Ethics Statement

This research was approved by the Office of Research Ethics at the University of Waterloo (Project 30395: Social Understanding in Children).

## Conflicts of Interest

The authors declare no conflicts of interest.

## Supporting information



Supporting Information

## Data Availability

Materials, data, and code from all experiments are available online at https://osf.io/a7nge/. These experiments were not preregistered.

## Endnotes

1 Strictly, these patterns are equally probable. However, adults see haphazard orders as more representative of randomness and often reject the idea that ordered structures could come about by chance (Kahneman and Tversky 1972). The findings on how children responded to changes in order (e.g., Friedman 2001; Newman et al. 2010) suggest they may have similar expectations.

2 The first six participants were tested with orders where the two trial types alternated (e.g., suspicious, expected, suspicious, and expected).

3 Asking the negative version of the question (i.e., “Which one didn't peek?”) could have introduced some difficulty for children. We included it, though, to rule out low‐level response strategies. For instance, although a bias to choose the character with the ordered arrangement would lead to correct responses for the question “Which one peeked?”, it would lead to incorrect responses for “Which one didn't peak?”. Asking the negative version of the question allowed us to check for this bias.
