# Supplementary material for: Who Peeked? Children Infer the Likely Cause of Improbable Success
Source: Dev Sci. 2024 Dec 20;28(1):e13598. doi: 10.1111/desc.13598 (PMC11660738; doi:10.1111/desc.13598)
Supplement: Supplementary file 1 — Supporting Information [file DESC-28-e13598-s001.docx]

**Supplementary Material for**

***Who peeked? Children infer the likely cause of improbable success***

Tables with mean response rates by age in years

**Experiment 1.** *Proportion of peeked responses*

| years | suspicious | expected |
| --- | --- | --- |
| 4 | 0.45 | 0.38 |
| 5 | 0.73 | 0.23 |
| 6 | 0.65 | 0.03 |
| 7 | 0.85 | 0.15 |

**Experiment 2.** *Proportion of peeked responses*

| years | proportion good | ordered | random |
| --- | --- | --- | --- |
| 4 | 2/10 yummy | 0.57 | 0.43 |
| 5 |  | 0.16 | 0.19 |
| 6 |  | 0.10 | 0.07 |
| 7 |  | 0.06 | 0.10 |
| 4 | 5/10 yummy | 0.53 | 0.57 |
| 5 |  | 0.44 | 0.41 |
| 6 |  | 0.33 | 0.27 |
| 7 |  | 0.58 | 0.26 |
| 4 | 8/10 yummy | 0.57 | 0.63 |
| 5 |  | 0.68 | 0.68 |
| 6 |  | 0.73 | 0.73 |
| 7 |  | 0.87 | 0.84 |

**Experiment 3.** *Choices of agent with ordered gumballs*

| years | peeked | didn't peek |
| --- | --- | --- |
| 5 | 0.71 | 0.45 |
| 6 | 0.60 | 0.33 |
| 7 | 0.83 | 0.30 |

**Experiment 4.** *Proportion of peeked responses*

| years | bad odds | good odds |
| --- | --- | --- |
| 5 | 0.67 | 0.46 |
| 6 | 0.79 | 0.46 |
| 7 | 0.92 | 0.45 |
